# Prevalence and associated factors of metabolic syndrome among pregnant Ethiopian women: a hospital-based cross-sectional study

**DOI:** 10.1038/s41598-024-65107-z

**Published:** 2024-06-23

**Authors:** Alemie Fentie, Getnet Fetene, Zeleke Kassahun, Sintayehu Ambachew

**Affiliations:** 1 Department of Clinical Chemistry, School of Biomedical and Laboratory Sciences, College of Medicine and Health Sciences, University of Gondar, P.O. Box 196, Gondar, Ethiopia; 2 Department of Medical Laboratory Science, College of Medicine and Health Sciences, Debre Tabor University, Debre Tabor, Ethiopia; 3 Adelaide Medical School, University of Adelaide, Adeliade, SA Australia

**Keywords:** Dyslipidemia, Hyperglycemia, Hypertension, Metabolic syndrome, Abdominal obesity, Pregnant women, Biochemistry, Diseases, Health care, Risk factors

## Abstract

Metabolic syndrome (MetS) poses a significant public health challenge globally, including in Ethiopia, with risks for both mothers and children. Unfortunately, there is limited data on MetS in pregnant Ethiopian women. This study aims to evaluate the prevalence and factors associated with MetS in this population. A cross-sectional study was conducted using a systematic random sampling technique. Data were collected through face-to-face interviews using a structured questionnaire adapted from the World Health Organization Steps Survey Tool for Non-communicable Diseases. About five ml of fasting peripheral blood samples were collected from each participant. The Beckman Coulter DXC 700 AU clinical chemistry analyzer was employed for lipid profile and glucose analysis. Subsequently, data were inputted into Epi Data and later exported to SPSS Version 20 for further analysis. Bivariable and multivariable binary logistic regression analyses were carried out, with a predefined level of statistical significance at p < 0.05. A total of 318 pregnant women were included in this study. The prevalence of MetS was 13.2% (95% CI: 9.7, 17.0) based on the American Heart Association/National Heart Lung and Blood Institute definition. The most prevalent components of MetS were elevated triglyceride levels, reduced high-density lipoprotein levels, and elevated blood pressure. Unhealthy sleep duration (AOR = 5.6, 95% CI (2.4, 13.1), p < 0.001), high daily salt intake (AOR = 4.2, 95% CI (1.8, 9.5), p = 0.001), and alcohol consumption [AOR = 4.2, 95% CI (1.6, 10.9), p = 0.003] were significantly associated with MetS. The study reported a high prevalence of MetS in pregnant Ethiopian women. Factors including alcohol, high salt intake, and sleep disturbances were associated with MetS. Policymakers might utilize this data to create targeted interventions and public health policies for MetS among pregnant women, focusing on nutrition, sleep, and alcohol consumption during pregnancy to safeguard maternal and fetal health.

## Introduction

Metabolic syndrome (MetS) is a cluster of conditions that occur together, increasing the risk of type 2 diabetes, heart disease and stroke. Conditions that constitute MetS includes high blood pressure, hyperglycemia, abdominal obesity, and dyslipidemia^1,2^. MetS and its components have been linked to lifestyle factors, such as alcohol use, cigarette smoking, sleeping length, sedentary behavior, and physical activity^3–5^.

Pregnancy is a risk factor for the development of MetS. This is a period of weight gain, hyperlipidemia, and insulin resistance^6^. This period induces substantial metabolic and hemodynamic shifts in a woman's physiology to support fetal growth. Triglyceride (TAG) levels surge to facilitate fetal development, while elevated high-density lipoprotein (HDL) aids effective lipid transport to the placenta. Low-density lipoprotein (LDL) levels may fluctuate due to hormonal changes. Insulin sensitivity decreases to ensure an ample glucose supply for the growing fetus, accompanied by increased glucose production by the liver. Postprandial glucose levels may vary and rise significantly. Prolonged persistence of these changes can lead to hypertensive disorders of pregnancy, such as gestational hypertension, preeclampsia, or eclampsia, as well as gestational diabetes^7^. These conditions impact both the mother and the fetus.

MetS affects one-quarter of the world's adult population and becomes more common as the People gets older. For instance, a study conducted in Turkey reported a 33.9% prevalence of MetS; women (39.6%) had a higher prevalence than men (28%)^8^. The prevalence of MetS also increases in Africa, ranging from 17 to 25%^9^. Similar findings have also been reported in some regions such as northwestern Nigeria (35.1%)^10^, Cape Town in South Africa (30.7%)^11^, Morocco (35.4%)^12^, Cameroon (32.45%)^13^, and Ethiopia (20.3%)^14^.

Studies have shown that MetS is common among females. Findings have indicated that 3–42% of pregnant women have MetS^15^. Similarly, heart disease was found to be the primary cause of maternal fatalities in the United Kingdom from 2010 to 2012, with an incidence of 2.25 deaths per 100,000 live births according to obstetrical surveillance^16^. Surveillance data in the United States (U.S.) indicated that the number of cardiovascular pregnancy-related mortalities increased from 348 per 100 000 live births from 1998 to 2005 to 423 in 2006 to 2010^17^.

Pregnant women exposed to MetS risk factors are reported to have a higher risk of adverse maternal and neonatal outcomes. These complications encompass abortion, preterm delivery, preeclampsia, gestational diabetes, gestational hypertension, postpartum hemorrhage, childbirth trauma, fetal abnormalities, low birth weight, intrauterine growth retardation, macrosomia, stillbirth, and mortality^18^. In addition, the accelerated growth of MetS has significant health implications that can increase the risk of cardiovascular disease, stroke, diabetes, and cancer. Beyond immediate concerns, maternal MetS is linked to long-term health risks for the child, including obesity and metabolic disorders. These all are economically cumbersome on a country due to increased healthcare costs, lost productivity, and the need for long-term medical care and support. Early deaths and disabilities caused by metabolic disorders are also currently challenging in both developed and developing countries. Early identification allows for targeted interventions, such as lifestyle modifications, to improve outcomes. In Ethiopia, there exists a notable scarcity of information concerning the prevalence and associated factors of MetS in pregnant women. Consequently, this study endeavors to fill this information gap by determining the prevalence and associated factors of MetS among pregnant women in the region. Hence, the outcomes of this research guide tailored healthcare strategies for promoting the well-being of both mothers and their offspring.

## Methods and materials

### Study design

A cross-sectional study was conducted at the University of Gondar comprehensive specialized hospital, Gondar, Ethiopia from March 14 to May 10, 2022.

### Sample size and sampling methods

The sample size for this study was calculated using the single population proportion formula: Z2 = p (1 − p) d2). Since the prevalence of MetS in Ethiopian pregnant women is unknown, the p-value was obtained from a study conducted on pregnant women in Angola (29.2%)^12^ with a confidence level of 95% and a marginal error (d) of 5%. In total, 318 pregnant women were included in this study, and the selection of participants was carried out through a systematic random sampling technique.

### Study participants

All pregnant women over 18 years old in their first to third trimesters were included in the study. Pregnant women who were severely ill, with known diabetes and hypertension (HTN) were excluded. Pregnant mothers who were in 2nd, and 3rd trimester, but came for their first visit or with reference were excluded because of the unavailability of pre-pregnancy weight, which was used to calculate the pre-pregnancy BMI. Additionally, pregnant women who were infected with HIV and seropositive for hepatitis B and C were also excluded by reviewing their medical charts and screening for first-trimester pregnant mothers. Infection with HIV and subsequent treatment with antiretroviral therapy and seropositivity for hepatitis B and C are associated with metabolic changes and affect the validity of the results. Excluding participants with hepatitis B and C from this study is essential to avoid potential confounding effects on metabolic parameters. These viral infections can induce liver inflammation, compromising liver function and influencing lipid profiles and glucose metabolism. The exclusion ensures a more accurate assessment of MetS components, isolating their specific impact.

### Data collection procedures

The data were collected using a pre-tested WHO STEPwise Approach to Surveillance (STEPS) for the NCDS, with some modification of the variables. This included information about the participants' sociodemographic status, medical status, lifestyle, and nutritional habits^1^. The questionnaire was first adopted in English and then translated into Amharic by a language expert. It contains sections for assessing demographics, lifestyle behaviors, and medical history records.

Data were collected by appropriately trained midwives and laboratory technologists who were supervised by a principal investigator. Sociodemographic, clinical, and behavioral characteristics and anthropometric data (weight, and height) were collected in the maternity ward (antenatal care clinic) through face-to-face interviews by trained midwifery professionals. In addition, antecubital venous blood specimens were obtained from the pregnant mother by a skilled laboratory technologist in the antenatal care laboratory ward.

Initially, informed consent was obtained, then anthropometric measurements such as blood pressure was taken using medium-sized digital measuring device (BP cuff) after resting for at least five minutes, and the actual pregnant weight was measured for first visit pregnant mothers using balance with weighed to the nearest 0.1 kg in light indoor clothing and bare feet. In addition, another weight for pregnant mothers was obtained during the first antenatal visit by referring to the registration log book.

The actual heights of the participants were measured using a movable stadiometer. Measurements were taken when the subject stood in an erect posture without shoes and recorded to the nearest 0.5 cm and the pre-pregnancy body mass index (BMI) of the mother was calculated using the formula (pre-pregnancy BMI = pre-pregnancy weight for 2nd and 3rd trimesters, and actual weight for 1st trimester divided by actual height squared)^1^.

### Diagnosis of MetS

For the diagnosis of MetS, the American Heart Association/National Herat Lung and Blood Institute definition was used. This definition has no absolute requirement for MetS components, population specificity, and a higher prevalence of MetS than other definitions (NCEP ATP III, IDF, and WHO) in pregnant mothers. According to this definition, MetS can be defined as any three or more of the following: elevated waist circumference (according to population- and country-specific definitions), plasma triglycerides ≥ 150 mg/dl or specific treatment for this lipid abnormality, blood pressure of 130/85 mmHg or therapy of previously diagnosed HTN, HDL cholesterol of less than 50 mg/dl, fasting plasma glucose of less than 100 mg/dl, or use of medication to treat increased glucose levels^2–4^.

### Laboratory procedures and processing

#### Pre-analytical test

Aseptically, five milliliters of venous blood were drawn from the antecubital fossa of the forearm following an overnight fast. Trained laboratory professionals skillfully collected the blood samples. The specimens were placed in serum separator test tubes and allowed to clot at room temperature for 10–20 min. Subsequently, the samples were transported to the laboratory for further analysis. To isolate the serum, the blood samples underwent centrifugation at 3000 rpm for five minutes. The lipid profile tests (Triglycerides, Total Cholesterol, HDL Cholesterol, LDL Cholesterol), along with fasting blood glucose levels, were examined utilizing a Beckman Coulter DXC 700 AU clinical chemistry analyzer.

#### Analytical test

The lipid profile tests and FBS test were measured at the University of Gondar Comprehensive Specialized Hospital in clinical chemistry laboratory section. After shipment and centrifugation, the samples were analyzed immediately after collection. However, when a delay was expected, the sample was stored in a refrigerator until analysis. The analysis of blood glucose was performed using two enzymes (hexokinase and glucose-6-phosphate dehydrogenase) and NAD+, which are reduced and absorb light at 340 nm measured photometrically. Lipid profiles were measured by sequential enzymatic reactions, and chromophores were added to measure the absorbance. Accelerators or precipitating agents used in the measurement of HDL and LDL in addition to chromophores and the absorbance of lipid profiles (total cholesterol, TAG, HDL, and LD) were measured photometrically at a specific wavelength. In addition, two levels (normal and pathological) of internal quality control (IQC) samples were run before serum samples were run using a Beckman Coulter DXC 700 AU machine. The control sample results were within the acceptable range for running the testing sample.

### Quality control measures for data

Before the actual data collection, the quality of the data was assured using a pre-tested questionnaire, which was completed at Maraki Health Center using 5% of the study participants. Data collectors were trained in data collection procedures, and awareness was given which included the relevance of the study, the objective of the study, confidentiality of the information, informed consent, and interview techniques.

The principal investigator reviewed the completed questionnaires at the end of the data collection for completeness. Corrective measures were taken in case of data collection difficulties, accordingly. Moreover, the data was carefully entered, cleaned, and checked for completeness, and missing values were treated accordingly.

The quality of laboratory result was maintained through proper sample collection, transportation, processing and storage and the laboratory analysis were performed by following manufactures instruction. The quality of analytical procedures of laboratory test was maintained by running normal and pathological quality control material before proceeding to run the test sample and the results of quality control material was interpreted based on WESTGARD rules.

Overall, ensuring the accuracy of blood pressure, weight, and height measurements had been pivotal in the reliability of a MetS study. For blood pressure measurements, regular calibration of devices and the use of proper cuff sizes were conducted. Participants were in a rested state, and multiple readings were averaged to ensure consistency. Weight measurements were done from calibrated scales. Participant consistency in clothing, and the removal of heavy items from pockets and footwear were employed. Height measurements employed by calibrated stadiometer, correct participant alignment, and attention to participant comfort. Staff involved in measurements underwent thorough training, and interobserver agreement checked consistently. Regular audits of procedures, equipment, and transparent reporting contributed to the overall quality control of measurements, and standardized protocols were followed consistently.

### Data processing and analysis

The pre-pregnant BMI, blood glucose, lipid profile, and blood pressure of pregnant women's results were recoded. The data was entered using Epi-data version 3.1. The data were cleared, edited, and checked for completeness, and then exported to SPSS version 20 for analysis. While organizing and cleaning the data, frequencies and percentages of the participants were calculated. The Pearson chi-square assumption was assessed for categorical variables. s. Univariate and multivariable binary logistic regression was used to measure the association between dependent and independent variables. In the present study, the process of selecting variables for logistic regression comprised a dual-step approach, integrating both univariate and multivariable methods. In the initial phase, univariate analysis was employed to explore individual predictor variables through the use of univariate logistic regression. Subsequently, significance testing was applied to evaluate the statistical significance of each variable's relationship with the outcome. Variables surpassing the significance threshold (P-value < 0.2) were then advanced to the next stage. The subsequent multivariable analysis involved constructing a model using the "enter" method, wherein the selected variables were introduced simultaneously. The significance of each variable within the multivariable model was rigorously assessed. The Hosmer–Lemeshow goodness of fit test with a p-value > 0.05 significant level was used to check the model fitness assumption. The level of statistical significance was set at a 95% confidence interval with a p-value of less than 0.05. Results were presented in the forms of tables, graphs, and narrative illustration.

### Ethical consideration

The study was conducted after ethical approval was obtained from the Research and Ethical Review Committee of the School of Biomedical and Laboratory Sciences, University of Gondar (Ref.No/SBMLS/208). The University of Gondar Comprehensive Specialized Hospital granted official permission, and consent was acquired from every study participant prior to commencing the actual data collection. Participants were informed about the risks and benefits of the study; their right to withdraw at any time, confidentiality was maintained using codes. All methods were implemented in accordance with University of Gondar ethical guidelines and regulations. Finally, abnormal findings were linked to the ANC clinic for further evaluation for better management and treatment. The patients/participants provided their consent to participate in this study. For those illiterate participants, the necessary explanation has been made and informed consent has been obtained from a parent and/or legal guardian for study participation.

## Results

### Socio-demographic characteristics of study participants

A total of 318 study participants with 100% response rate were included in this study. Of these, majority of the participants were found to be aged 20–34, 245 (77%) with a mean age of 28.6 ± 5.4. Most of the participants 272 (85.54%) live in urban areas (Table 1).

**Table 1:** Sociodemographic characteristics of pregnant women at the University of Gondar Comprehensive Specialized Hospital, 2022 (n = 318).

Variables	Categories	n (%)
Pregnancy trimesters	First	106 (33.3)
	Second	106 (33.3)
	Third	106 (33.3)
Age	< 20	25 (7.9)
	20–34	245(77.0)
	35–39	33 (10.4)
	≥ 39	15 (4.7)
Marital status	Single	21 (6.6)
	Married	293 (92.14)
	Divorced	2 (0.63)
	Widowed	2 (0.63)
Education status	Unable to read & write	51 (16.04)
	Complete primary school	48 (15.05)
	Complete secondary school	107 (33.65)
	Higher education	112 (35.22)
Residence	Urban	272 (85.54)
	Rural	46 (14.46)
Occupation	Government employed	90 (28.30)
	NGO employed	25 (7.86)
	Private employed	44 (13.84)
	Student	31 (9.75)
	Housewife	101 (31.76)
	Other	27 (8.49)
Family monthly income	< 1600 ETB	107 (33.65)
	1600–2600 ETB	40 (12.58)
	2601–3600 ETB	56 (17.61)
	> 3600 ETB	115 (36.16)

*ETB* Ethiopian birr, NGO non-governmental organization, other occupation (farmers, merchants, etc).

### Clinical characteristics of study participants

Among the study participants, 310 (97.48%) were non-smokers, 192 (60.38%) of the participants had taken alcohol before and during pregnancy, 294 (92.5%) of study participants did not have regular exercise, 215 (67.6%) with normal sleep duration and 225 (71.21%) utilize standard dietary salt (Table 2).

**Table 2:** Frequency distribution of lifestyle, behavioral, and clinical factors of pregnant women at the University of Gondar Comprehensive Specialized Hospital, Gondar, Ethiopia, 2022 (n = 318).

Variables	Categories	n (%)
Have you ever smoked any tobacco products	Yes	8 (2.52)
	No	310 (97.48)
Alcohol consumption before and during pregnancy	No	126 (39.62)
	Yes	192 (60.38)
Did you use sugary foods and drinks	No	96 (30.19)
	Yes	222 (69.89)
Dietary sugar consumption	Standard	209 (94.14)
	Above standard	13 (5.86)
Frequency of eating fruits/week	Every day	6 (1.88)
	Never	256 (80.50)
	Sometimes	56 (17.6)
Frequency of eating Vegetables /week	Every day	13 (4.10)
	Never	202 (63.52)
	Sometimes	103 (32.39)
Did eat meat in the past 12	Yes	297 (93.4)
	No	21 (6.6)
Daily salt consumption	Standard users	225 (71.21)
	Above the standard	91 (28.79)
Fat/oil type that you use at home	Nut oil	13 (4.10)
	Butter	15 (4.71)
	Vegetable oil	142 (44.65)
	Sunflower	139 (43.71)
	Other	9 (2.83)
Habit of eating outside home	Non-users	279 (87.7)
	Users	39 (12.3)
Regular exercise	No	294 (92.5)
	Yes	24 (7.55)
Physical activity level	Moderate	11(3.5)
	In active	105 (33.02)
	Light	202 (63.5)
Sleep duration status	Normal	215 (67.6)
	Abnormal	103 (32.4)
Gravidity	Primigravida	157 (39.37)
	Multigravida	161 (50.63)
Parity	Nulliparous/primiparous	171 (53.8)
	Multiparous	147 (46.23)
Abortion	No	260 (81.76)
	Yes	58 (18.24)
Family history of HTN/DM	No	300 (94.34)
	Yes	18 (5.66)
Habit of obstructive sleep	No	307 (96.54)
	Yes	11 (3.46)

*DM* diabetes mellitus, *HTN* hypertension.

### Prevalence of MetS among pregnant women

**Figure 1:**
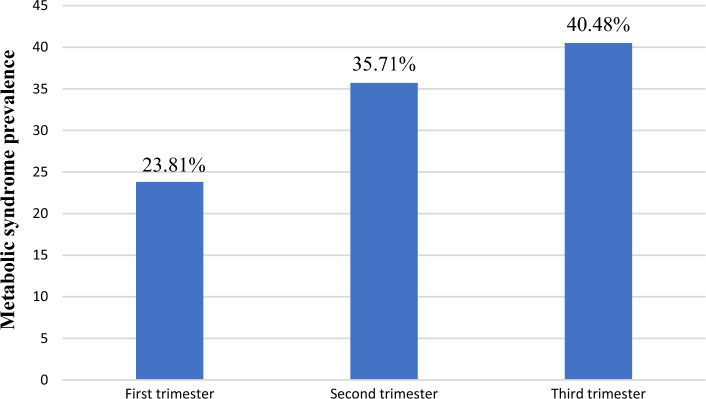
Proportion of MetS in pregnancy trimester among overall prevalence at the University of Gondar Comprehensive Specialized Hospital, Gondar, Ethiopia, 2022.

American Heart Association /National Heart Lung and Blood Institute of MetS diagnostic criteria was used in this study. Based on this definition, the prevalence of MetS was showed 42 (13.2%) (95% CI: (9.7, 17.0)). Based on trimester of pregnancy, the prevalence of MetS was 10 (3.14%), 15 (4.72%) and 17 (5.35%) in the first, second and third trimester, respectively. This accounts for 23.81%, 35.71%, and 40.48% of the overall MetS among pregnant women, respectively (Fig. 1).

### Prevalence of MetS components among pregnant women

**Figure 2:**
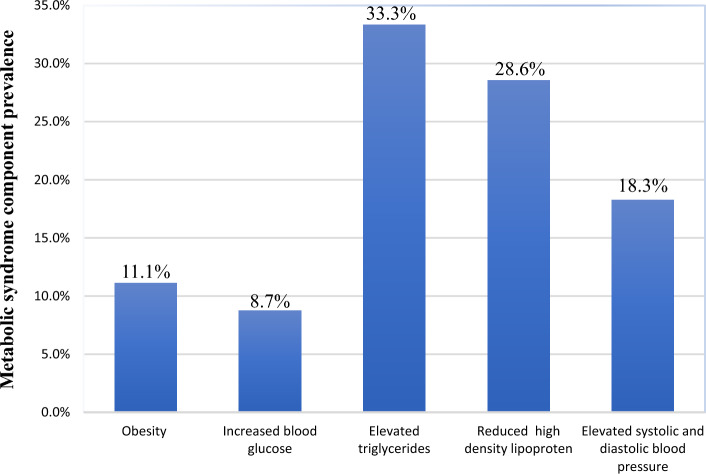
MetS components among pregnant women at the University of Gondar Comprehensive Specialized Hospital, Gondar, Ethiopia, 2022.

Based on NHLBI, in this study, the most frequent component of MetS was high level of TAG (33.3%), low level of HDL (28.6%), elevated FBS (8.7%), DBP/SBP (18.3%), and elevated pre-pregnancy BMI (11.1%) as seen in Fig. 2.

### Determinant factors of MetS among pregnant women

Alcohol intake before pregnancy and during pregnancy (p = 0.002), cigarette smoking (p = 0.057), eating non-cooked foods at home (p = 0.005), types of oil used at home(p = 0.016), excessive daily salt consumption (p ≤ 0.001)), physical activity level (p = 0.155), sleep disturbance (P  ≤ 0.001), gravida (p = 0.001) parity (p = 0.001), and family history of HTN, & DM (p = 0.014), and obstructive sleep (p = 0.175) were candidate variables for multivariable binary logistic regression. Among the predictor variables analyzed in multivariable binary logistic regression, alcohol intake before and during pregnancy [AOR = 4.2, 95% CI (1.6, 10.9), p = 0.003], high daily salt consumption [AOR = 4.2, 95% CI (1.8, 9.5), p = 0.001] and sleep disturbance [AOR = 5.6, 95% CI (2.4, 13.1), p =  < 0.001] were statistically significant with MetS among pregnant women as seen in (Table 3).

**Table 3:** Bivariable and multivariable binary logistic regression of MetS with lifestyle, behavioral and clinical factors among pregnant women at the University of Gondar Comprehensive and Specialized Hospital, Gondar, Ethiopia, 2022 (n = 318).

Variables	Categories	MetS (N = 318)		COR 95% CI	AOR 95% CI	P-value
		Yes n (%)	No n (%)			
Smoking	Non-smokers	39 (12.26)	274 (86.16)	1	1	–
	Past smokers	3 (0.94)	5 (1.57)	4.169 (0.958, 18.136)	2.799 (0.276, 28.412)	0.384
Alcohol intake before, and during pregnancy	No	7 (2.2)	119 (37.41)	1	1	–
	Yes	35 (11.00)	157 (49.37)	3.8 (1.6, 8.8)	4.2 (1.6, 10.9)	0.003*
Type of oil /fat used mostly at home	Nut oil	3 (0.94)	10 (3.14)	1	1	–
	Butter	6 (1.88)	9 (2.83)	2.222 (0.426, 11.603)	1.113 (0.119, 10.418)	0.925
	Vegetable oil	21 (6.6)	121 (38.05)	0.579 (0.147, 2.279)	0.684 (0.103,4.568)	0.695
	Sunflower	11 (3.46)	128 (40.25)	0.286 (0.069, 1.197)	0.487 (0.069, 3.412)	0.469
	Other	1 (0.31)	8 (2.51)	0.417 (0.036, 4.813)	0.264 (0.014, 5.004)	0.375
Daily salt intake	Standard-users	18 (5.69)	207 (65.51)	1	1	–
	Above-standard	24 (7.59)	67 (21.20)	4.0 (2.05, 7.81)	4.2 (1.8, 9.5)	0.001**
Eating foods not cooked at home	Non-users	31 (9.75)	248 (77.99)	1	1	–
	Users	11 (3.45)	28 (8.81)	3.143 (1.425, 6.933)	2.246 (0.765, 6.598)	0.141
Physical activity level	Moderate	2 (0.60)	9 (2.82)	1	1	–
	Inactive	19 (5.97)	86 (27.04)	0.994 (0.199, 4.977)	2.11 (0.302, 14.740)	0.451
	Light	21 (6.60)	181 (56.92)	0.522 (0.106, 2.579)	1.73 (0.251, 11.975)	0.577
Sleep duration status	Normal	12 (3.77)	203 (63.84)	1		
	Abnormal	30 (9.43)	73 (22.96)	6.95 (3.4, 14.3)	5.6 (2.4, 13.1)	< 0.001**
Gravidity	Primigravida	10 (3.15)	144 (45.28)	1	1	–
	Multigravida	32 (10.06)	132 (41.51)	3.491 (1.652, 7.378)	1.630 (0.392, 6.781)	0.502
Parity types	Null/primiparous	12 (3.77)	157 (49.37)	1	1	–
	Multiparous	30 (9.43)	119 (37.42)	3.298(1.621, 6.713)	2.139 (0.535, 8.547)	0.282
Family history of HTN and DM	No	36 (11.32)	264 (83.01)	1	1	–
	Yes	6 (1.88)	12 (3.77)	3.667 (1.296, 10.374)	1.699 (0.428, 6.741)	0.451
Obstruction during sleep	No	39 (12.26)	268 (84.27)	1	1	–
	Yes	3 (0.94)	8 (2.52)	2.577 (0.656, 10.129)	2.868 (0.362, 22.698)	0.318

*AOR* adjusted odd ratio, *COR* crude odd ratio, *CI* confidence interval, *DM *diabetes mellitus, *HTN* hypertension, *MetS* metabolic syndrome, *UOGCSH *University of Gondar Comprehensive Specialized Hospital.

N.B: * significant at p ≤ 0.05 level, ** highly significant at p < 0.05 level, 1 = the reference group. Other type of oil (soya bean oil, nugget oil, olive oil etc).

## Discussion

According to the American Heart Association/ National Heart Lung and Blood Institute (AHA/NHLBI) definition, the overall prevalence of MetS in the present study was 13.2% (95% CI (9.7–17.0)). This study aligns with the reported prevalence in Angola (12.6%)^5^, India (12%)^6^ , and a multi-center study conducted in New Zealand, Australia and UK (12.4% and 12.3%)^7,8^. However, the prevalence observed in the current study is higher than that reported in studies conducted in Cameroon (7%)^9^, Brazil (3%)^10^, India (8%)^11^, and Sri Lanka (5.6%, 5.4%, 4.2%, and 3% based on AHA/NHLBI, IDF, NCEP-ATP III, and WHO diagnosis criteria, respectively)^12^. In contrast, the prevalence observed in the current study was also lower than that reported in studies conducted in Angolan (36% and 29.2% (based on Joint interim analysis and NCEP ATPIII)^5^, Cameroon (17.88%)^13^ and a multicenter study in Australia, New Zeeland, and UK (20%)^14^. The variations in the results may stem from differences in gestational age, with some studies focusing on specific pregnancy trimesters. For instance, the study in Brazil, India, and the multicenter study in Australia, New Zealand, and the UK specifically included second-trimester pregnant women^7,8,10,11,14^, and first trimester pregnant women were used in in Sri Lanka^12^. Indeed, the difference in the use of different MetS diagnostic criteria, the sample size and laboratory diagnostic methods used may cause the variation of MetS prevalence.

Furthermore, the specific MetS components may account for variations in the prevalence of MetS. For instance, in Angolan pregnant women, the major component of MetS was fasting blood glucose in the joint interim analysis and central obesity in NCEP ATPIII, but high TAG and reduced HDL level was the most frequent MetS components in the current study. Since central obesity and hyperglycemia are the main triggering factors for the pathophysiology of MetS^5^. Insulin resistance and visceral adiposity contribute to dyslipidemia and endothelial dysfunction, with MetS seemingly necessitating a combination of a metabolic predisposition to insulin resistance and obesity^15^.

The prevalence of TAG in this study was 33.3%. This study was almost similar with the reports in Brazil (31.5%)^10^, a multicenter study in Australia, New Zealand and UK (29.15%) and Angola (28.99%)^5,8^. However, the current study was lower than the findings reported in India (55%, 44%)^6,11^ and higher than other studies done in Cameroon (3.7%)^9^, Sri Lanka (6.9%)^12^ and a multi-center study in Australia, New Zealand, and UK (24.52%, 25%)^7,14^. The observed discrepancy could be linked to variations in lifestyle factors, ethnicity, genetic influences, and differences in gestational age.

The prevalence of reduced HDL was observed to be 28.6%, showing a similarity to findings in Brazil (31.5%)^10^. The current finding was lower as compared to the studies reported in Cameroon (66%)^13^, India (56%)^11^ and Sri Lanka (52.5%)^12^. Conversely, it was higher compared to results in Angola (11.9%)^5^, Cameroon (16.3%)^9^, India (23%)^6^, and a multi-center study in Australia, New Zealand, and UK) (5.6%, 19.61%, 11.58%)^7,8,14^. The variation may be attributed to differences in gestational age, as HDL concentration tends to decrease with advancing pregnancy^16^. Additionally, genetic, ethnic, and lifestyle factors may contribute to the observed differences in prevalence. The prevalence of elevated DBP/SBP in this study was 18.3%, which was almost consistent report in India (20.5%)^6^.While the current study was lower as compared to the reports in Angola (29.5%)^5^. While a higher prevalence of elevated BP was obtained in the current study as compared to the study done in Sri Lanka (2.3%)^19^, a multi-center study in Australia, New Zealand, and UK (3.69%, 3.65%) (45, 46). The observed difference could be ascribed to variations in ethnicity, genetics, lifestyle factors, and the potential influence of gestational age. Existing evidence suggests that blood pressure (BP) tends to exhibit significant variations as gestational age progresses^16^.

In this study MetS in pregnant mothers was positively associated with alcohol consumption before and during pregnancy (AOR = 4.2, 95% CI (1.6, 10.9), p = 0.003)). This finding was consistent with the findings obtained from the study done in Cameroon, multicenter (Australia, New Zealand, and UK), and other previous studies^8,13,17–19^. Existing evidence suggests that excessive alcohol consumption leads to the accumulation of fat, possibly due to its nutritional content and its impact on suppressing lipid oxidation. Moreover, alcohol is implicated in oxidative stress, which can further influence insulin sensitivity^18,19^.

Unhealthy sleep duration (short and long) was also associated with MetS in pregnant women (AOR = 5.6, 95% CI (2.4, 13.1), p =  < 0.001) in the present study. This result was consistent with the findings obtained from a particular study done in Cameroon, India and other studies^11,17,20–22^. Scientific data supported that sleep deprivation and circadian disruption are linked to metabolic dysregulation, which may contribute to weight gain, obesity, and type 2 diabetes. Sleep disturbances can potentially trigger MetS by influencing the timing and quantity of food intake, disrupting energy balance, promoting inflammation, and impairing glucose tolerance and insulin sensitivity^23^. Delayed initiation of sleep, particularly after midnight, results in the suppression of the most significant growth hormone pulse and disrupts the secretion of cortisol hormone. Cortisol, often referred to as the "stress hormone," plays a crucial role in the body's response to stress, regulation of metabolism, and maintenance of homeostasis. However, disrupted cortisol secretion is associated with MetS^20^.

In the present study, excessive dietary salt intake was positively associated with the prevalence of MetS (AOR = 4.2, 95% CI (1.8, 9.5), p = 0.001). Health care providers advised that adults should consume no more than 5 g (a little under a teaspoon) of salt per day, according to the WHO in 2015^24^. Consumption of salt beyond the standard leads to kidney work overload, resulting in excess sodium and water retention. A high-salt diet worsens HTN and impairs the response of the vasculature to nitric oxide (NO) and promotes elevated BP^25^. Moreover, changes in metabolic pathways related to sodium sensitivity may bring about obesity and insulin resistance^26^.

As a limitation to this study, assessing MetS in pregnant women using weight and waist circumference has been a challenge. Physiological changes during pregnancy, gestational weight gain, and fluid retention impact the accuracy of these measures. Interpreting waist circumference is challenging due to abdominal expansion. This could lead to non-differential misclassification of MetS. The dynamic nature of metabolic changes is not fully captured, and establishing universal cutoff values is difficult due to population variability. To enhance a better assessment, considering additional biomarkers and accounting for individual variability is crucial for a more comprehensive understanding of metabolic health during pregnancy.

This study highlights a noteworthy prevalence of MetS among pregnant women, accounting 13.21%. High frequency of MetS components were recorded including high TAG levels, low HDL, and elevated blood pressure. Factors contributing to this prevalence include pre-pregnancy conditions, alcohol intake, excessive dietary salt consumption, and suboptimal sleep durations. Understanding these factors is crucial for developing effective interventions to mitigate MetS risk in pregnant women.

## Data Availability

The data is available on the primary and corresponding author up on request.

## Abbreviations

AHA: American Heart Association
BMI: Body mass index
DBP: Diastolic blood pressure
DM: Diabetes mellitus
HDL: High-density lipoprotein
HIV: Human immunodeficiency virus
HTN: Hypertension
IDF: International Diabetic Federation
JIS: Joint interim statement
LDL: Low-density lipoprotein
MetS: Metabolic syndrome
NCEP ATP: National Cholesterol Education Program Adult Treatment Panel
NHLBI: National Heart Lung and Blood Institute
TAG/TG: Triacyl glycerol/triglycerides
WHO: World Health Organization
